# Estimating the Impact of COVID-19 on the PM_2.5_ Levels in China with a Satellite-Driven Machine Learning Model

**DOI:** 10.3390/rs13071351

**Published:** 2021-04-01

**Authors:** Qiulun Li, Qingyang Zhu, Muwu Xu, Yu Zhao, K. M. Venkat Narayan, Yang Liu

**Affiliations:** 1 Gangarosa Department of Environmental Health, Rollins School of Public Health, Emory University, Atlanta, GA 30322, USA; 2 School of The Environment, Nanjing University, Nanjing 210023, China; 3 Hubert Department of Global Health, Rollins School of Public Health, Emory University, Atlanta, GA 30322, USA

**Keywords:** PM_2.5_, air pollution, COVID-19, MAIAC AOD, random forest, machine learning, remote sensing, China

## Abstract

China implemented an aggressive nationwide lockdown procedure immediately after the COVID-19 outbreak in January 2020. As China emerges from the impact of COVID-19 on national economic and industrial activities, it has become the site of a large-scale natural experiment to evaluate the impact of COVID-19 on regional air quality. However, ground measurements of fine particulate matters (PM_2.5_) concentrations do not offer comprehensive spatial coverage, especially in suburban and rural regions. In this study, we developed a machine learning method with satellite aerosol remote sensing data, meteorological fields and land use parameters as major predictor variables to estimate spatiotemporally resolved daily PM_2.5_ concentrations in China. Our study period consists of a reference semester (1 November 2018–30 April 2019) and a pandemic semester (1 November 2019–30 April 2020), with six modeling months in each semester. Each period was then divided into subperiod 1 (November and December), subperiod 2 (January and February) and subperiod 3 (March and April). The reference semester model obtained a 10-fold cross-validated R^2^ (RMSE) of 0.79 (17.55 μg/m^3^) and the pandemic semester model obtained a 10-fold cross-validated R^2^ (RMSE) of 0.83 (13.48 μg/m^3^) for daily PM_2.5_ predictions. Our prediction results showed high PM_2.5_ concentrations in the North China Plain, Yangtze River Delta, Sichuan Basin and Xinjiang Autonomous Region during the reference semester. PM_2.5_ levels were lowered by 4.8 μg/m^3^ during the pandemic semester compared to the reference semester and PM_2.5_ levels during subperiod 2 decreased most, by 18%. The southeast region was affected most by the COVID-19 outbreak with PM_2.5_ levels during subperiod 2 decreasing by 31%, followed by the Northern Yangtze River Delta (29%) and Pearl River Delta (24%).

## 1. Introduction

In December 2019, a cluster of patients infected with a novel betacoronavirus was reported in Wuhan, China [1]. The isolated virus, named SARS-CoV-2 [2], is highly infectious and rapid human-to-human transfer has been confirmed widely [1,3,4]. The coronavirus disease 2019 (COVID-19) posed global challenges for public health. As of 23 January 2020, one day before the Chinese New Year, at least 1975 cases had been reported [5] since the first hospitalized patient on 12 December. In order to contain the outbreak, China raised its national public health response to the highest state of emergency and implemented massive public health interventions. Wuhan, the epicenter of the outbreak, was the first city locked down and its neighboring cities started control thereafter. The central and local governments coordinated and implemented stringent social distancing measures and mobility restrictions [6]. The draconian interventions included isolation of suspected and confirmed cases, banning of public gatherings and close of schools as well as unnecessary commercial operations. In addition, the governments prohibited travelling in and out of cities and suspended public transport by bus and subway [7].

In addition to containing the spread of COVID-19, the lockdown and traffic restriction measures may have additional health benefits. In previous evaluations, declines of fine particulate matters (PM_2.5_) and other anthropogenic air pollutants such as ozone and nitrogen dioxide had been observed. For example, from one month before and after the lockdown, Wuhan showed a decline of 36.9% in PM_2.5_ levels compared with corresponding periods from 2015–2019 [8]. He et al. found similar results in which PM_2.5_ levels in locked-down cities were brought down by 7.05 μg/m^3^ relative to the previous year [9]. PM_2.5_ is a major public health concern and its exposure has been linked to many health issues. Previous studies suggested strong positive relationships between PM_2.5_ exposure and excess mortality [10], cardiovascular disease [11-13], respiratory symptoms [14], adverse pregnancy outcomes [15,16], influenza-like illness risk [17] and others. Recent studies found that each 1 μg/m^3^ increase of long-term exposure to PM_2.5_ is associated with 8% increase in the COVID-19 mortality rate [18].

Accurate estimation of PM_2.5_ concentrations is a prerequisite to quantify health benefits of reduced air pollution from COVID-19 control measures. China was a suitable study domain for air pollution research under the pandemic for two reasons. It was the first country attacked by COVID-19 epidemic and it implemented stringent countermeasures to prevent infections. In addition, PM_2.5_ is a major public health burden in China, with estimates suggesting that the air pollution contributes to 1.6 million deaths/year (0.7–2.2 million deaths/year at 95% CI), roughly 17% of the total deaths [19]. If COVID-19 control measures substantially improved the air quality in China, a greater magnitude of implied health benefits will be observed in China than in countries with lower initial air pollution levels. While many studies provided changes of PM_2.5_ levels during the COVID-19 pandemic, they used ground-based measurements [8,9,20,21]. Ground-based central PM_2.5_ monitors in the regulatory network in China are unable to capture the fine scale patterns of exposure and they lack coverage in rural areas. In addition, previous studies conducted in China were at city-scale or only focused on urban areas [8,9,22,23]. There was a lack of spatiotemporally resolved PM_2.5_ estimates during the COVID-19 outbreak and a comprehensive assessment of PM_2.5_ levels.

In this study, we developed a machine learning model with a method, the random forest algorithm, and used a large number of datasets as predictor variables. We validated the model with 10-fold cross-validation and predicted reliable daily PM_2.5_ concentrations over 5 km × 5 km grid cells across China during the study period, with a total length of 363 days. We estimated the impact of COVID-19 pandemic on PM_2.5_ levels by comparing concentrations in different periods. Our spatiotemporally resolved daily PM_2.5_ estimates allow epidemiologists to further quantify the health benefits of reduced air pollution with higher accuracy.

## 2. Materials and Methods

### 2.1. Study Area and Time Periods

**Figure 1.**
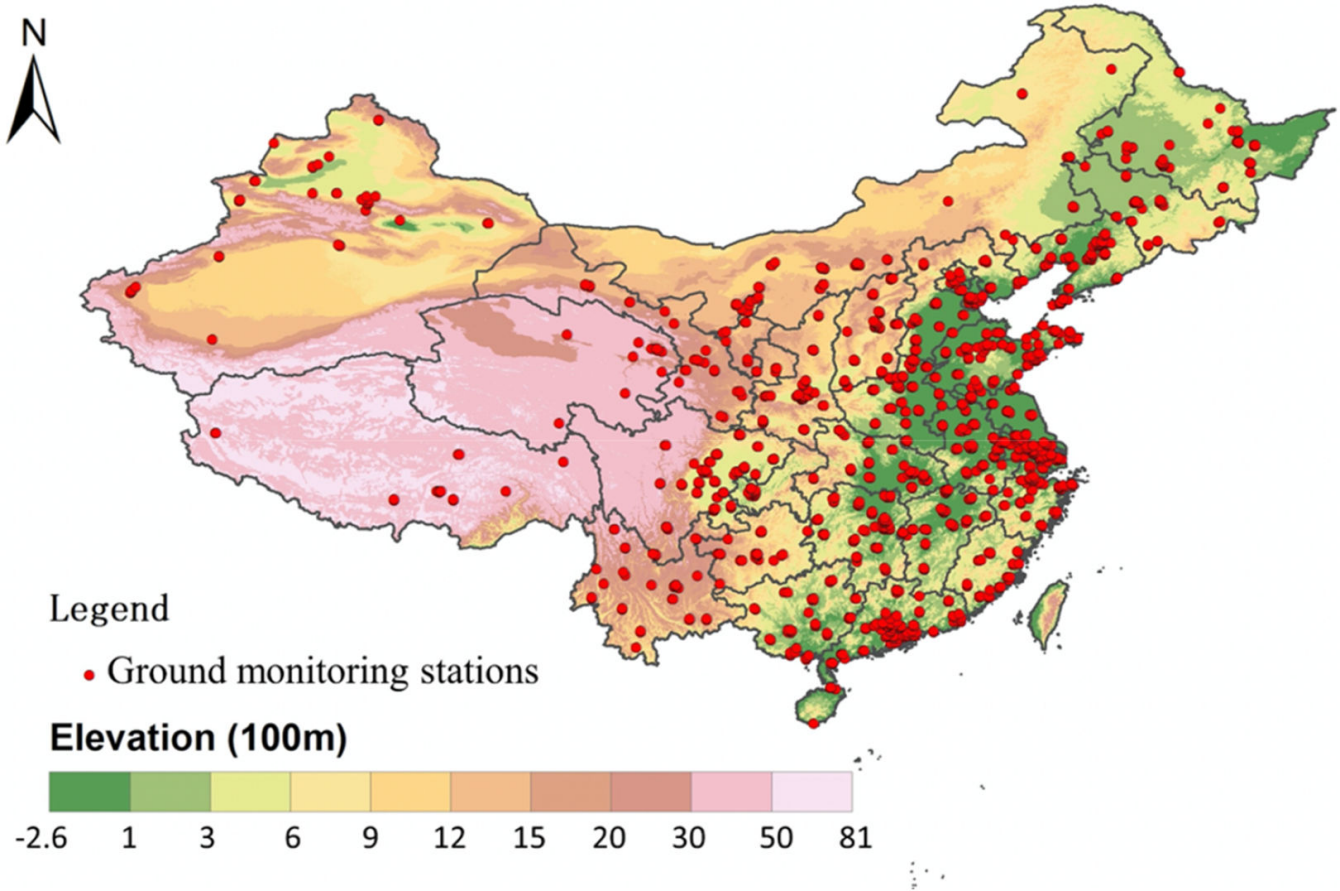
Map of the study domain. Ground monitoring stations are shown as red spots. China map with province outlines was downloaded from http://www.resdc.cn/, accessed on 20 March 2021, and the elevation data were obtained from the Advanced Spaceborne Thermal Emission and Reflection Radiometer (ASTER) Global Digital Elevation Model (GDEM) version 3.

The study domain includes mainland China, Hong Kong and Taiwan (Figure 1). It covers an area of 9.597 million km^2^ and has a population of approximately 1.4 billion (http://data.stats.gov.cn/index.htm, accessed on 20 March 2021). We created a 0.05° (approximately 5 km) resolution modeling grid covering this study area for data integration, with a total of 399,513 grid cells. Our study period consists of a reference semester, from 1 November 2018 to 30 April 2019, and a pandemic semester, from 1 November 2019 to 30 April 2020. Each period was then divided into subperiod 1 (November and December), subperiod 2 (January and February) and subperiod 3 (March and April). The pandemic subperiod 2 was considered as the COVID-19 outbreak period due to high number of cases reported and the implementation of stringent control measures [6,7].

### 2.2. Data

A summary of the datasets adopted to develop our spatial-temporal random forest models in this study is shown in Table S1 and described in detail below.

#### 2.2.1. PM2.5 Monitoring Data

Hourly PM_2.5_ concentration measurements were published by the China National Environmental Monitoring Center (CNEMC, http://www.cnemc.cn, accessed on 20 March 2021) and were downloaded from PM25.in (http://pm25.in/, accessed on 20 March 2021), with 1534 air monitoring sites in mainland China from 2018 to 2020. We obtained PM_2.5_ measurements in Hong Kong and Taiwan from the Hong Kong environmental protection department (http://epic.epd.gov.hk/, accessed on 20 March 2021) and the Taiwan environmental protection agency (http://taqm.epa.gov.tw/, accessed on 20 March 2021), respectively. We calculated daily averaged PM_2.5_ at every monitoring station and assigned monitoring stations to our modeling grid. PM_2.5_ within the same grid cell was averaged, and we got as many as 1252 grid cells with PM_2.5_ measurements.

#### 2.2.2. MAIAC AOD Data

We downloaded Terra (overpass at 10:30 local time) and Aqua (overpass at 13:30 local time) multi-angle implementation of atmospheric correction (MAIAC) AOD retrievals at 0.55 μm wavelength at 1 km resolution from NASA EarthData (https://search.earthdata.nasa.gov, accessed on 20 March 2021). Aerosol optical depth (AOD) is the measure of light extinction due to the presence of aerosols in the atmospheric column [24,25]. Many studies have investigated the relationship between AOD and ground PM_2.5_ measurements and AOD has been widely applied in PM_2.5_ modeling [26,27]. MAIAC is an advanced algorithm used to retrieve daily atmospheric properties at 1 km resolution based on the measurements of the Moderate Resolution Imaging Spectroradiometer (MODIS). It uses time series analysis and a combination of pixel- and image-based processing to improve accuracy of cloud detection, aerosol retrievals and atmospheric correction [28].

#### 2.2.3. Meteorological Parameters

Meteorological parameters during the study period were obtained from the Goddard Earth Observing System Data Assimilation System GEOS-5 Forward Processing (GEOS 5-FP) at a 0.25° latitude × 0.3125° longitude resolution (Lucchesi 2013). The temporal resolution was hourly for two-dimensional products and thrice-hourly for three-dimensional products (Lucchesi 2013). The meteorological data were downscaled to a 5 km grid cell by inverse distance weighting. We averaged hourly and thrice-hourly GEOS 5-FP data from 10:00 to 16:00 local time, respectively, to get the average weather conditions between Aqua and Terra overpass time. The full list of 15 meteorological variables used in this study can be found in the supplementary material (Table S1).

#### 2.2.4. Land Use Data

We obtained the land cover data from the ESA Climate Change Initiative (CCI) global land cover at 300 m resolution (https://www.esa-landcover-cci.org, accessed on 20 March 2021). The elevation data were extracted from the Advanced Spaceborne Thermal Emission and Reflection Radiometer (ASTER) Global Digital Elevation Model (GDEM) version 3 at 30 m resolution (https://asterweb.jpl.nasa.gov/gdem.asp, accessed on 20 March 2021). In addition, we obtained the population density data from the LandScan Global population database (http://landscan.ornl.gov/, accessed on 20 March 2021) at 1 km resolution.

### 2.3. Data Integration

All predictors with various spatial resolutions were fitted into our 5 km modeling grid. The MAIAC Aqua and Terra AOD data were processed and matched to the 5 km modeling grid using nearest neighbor approach in Python (version 3.7.6). The average of Aqua and Terra measurements was calculated for daily PM_2.5_ predictions. For days without Terra data, Aqua data were used to estimate the missing Terra values [29]. We multiplied Aqua values by an adjustment factor to account for diurnal variations [30]. Then we conducted aggregation of the MAIAC AOD dataset by averaging multiple AOD pixels within the same modeling grid. For the meteorological fields, the inverse distance weighting method was employed using R software (version 3.6.3). For each grid cell, the population density, elevation and land cover data were processed using ArcGIS software (version 10.7.1).

### 2.4. Spatial Cluster Analysis

**Figure 2.**
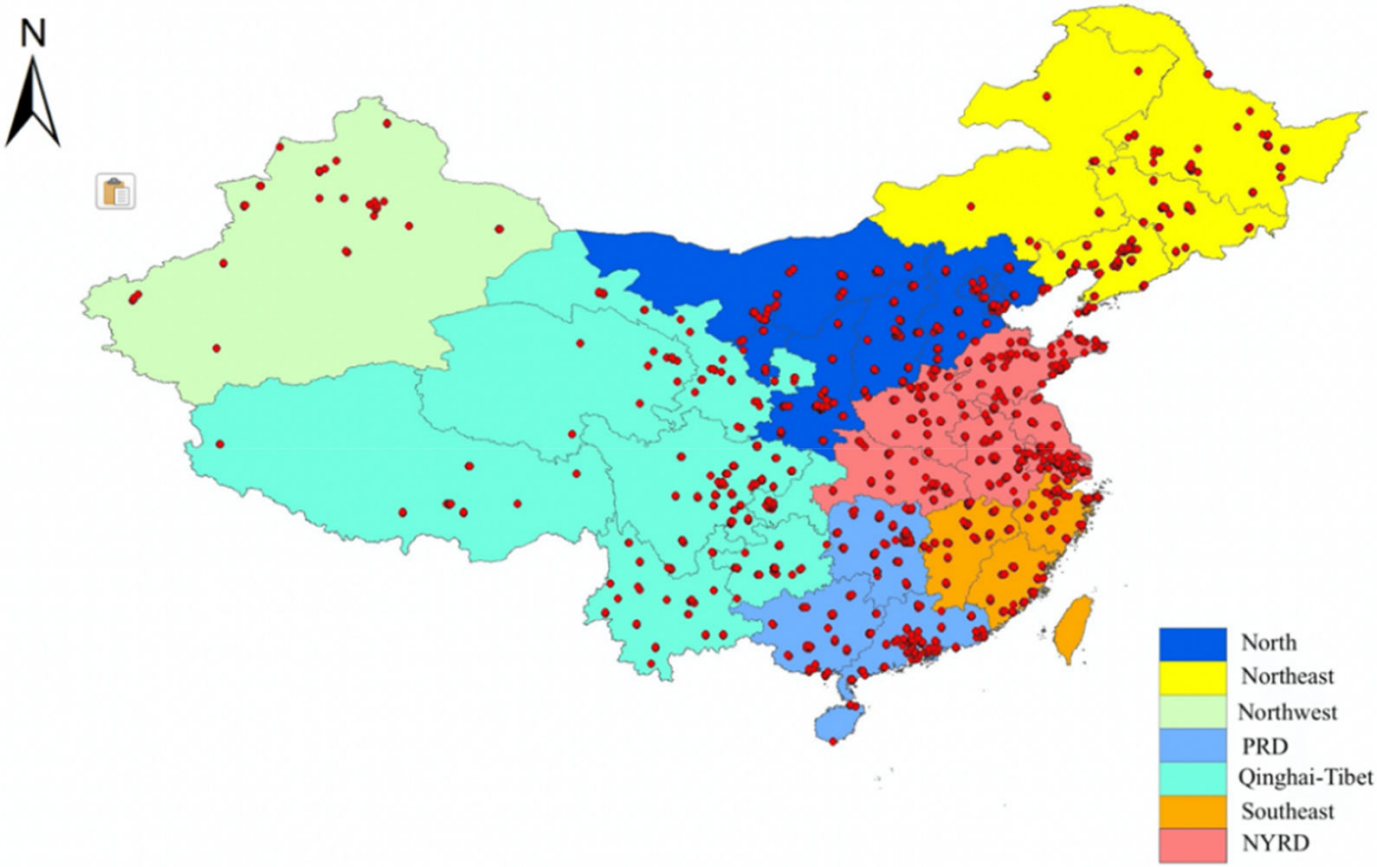
Seven subregions covering the study domain and spatial distribution of fine particulate matters (PM_2.5_) monitoring sites involved in this study. ArcGIS software was used for spatial cluster analysis (version 10.7.1).

Our study domain was divided into seven subregions to better characterize geographical and anthropogenic emission variations: Northeast, North, Northwest, West, Northern Yangtze River Delta (NYRD), Southeast and Pearl River Delta (PRD) (Figure 2). We fitted the same model structure in each cluster and used spatial prediction pattern for discussion. The creation of subregions followed the method of Xiao et al. [31] but we aligned clusters more closely along provincial boundaries. The Northeast subregion consisted of three northeastern provinces, i.e., Heilongjiang, Jilin and Liaoning, as well as eastern Inner Mongolia, where there is a long winter/heating season and large presence of heavy industry including iron and steel industry, machinery manufacturing, automobile manufacturing, oil processing, etc. [32-34]. The North China Plain and western Inner Mongolia constituted the North cluster, characterized by its coal consumption and stagnant weather, with weak wind and relatively low boundary layer height [35]. Xinjiang province constituted the Northwest cluster, characterized by substantial dust emissions from the Taklamakan Desert. Tibet plateau, Qinghai, Sichuan, Yunnan and Gansu province constituted the West subregion with a high altitude and low population density. The Yangtze River Delta was divided into two subregions: the northern Yangtze River Delta (NYRD) with central heating in winter and the relatively warm south without central heating (Southeast). The Pearl River Delta (PRD) was another subregion, located on the coast with warm weather. The PRD and Southeast subregions also produce more hydroelectricity than other regions. The subregion map was fitted into our 5 km modeling grid and each grid cell was assigned to a subregion.

### 2.5. PM_2.5_ Modeling

After integrating all datasets, we developed two separate random forest models to predict daily PM_2.5_ concentrations for reference year and pandemic year, respectively. Random forest models generated rankings of variable importance, which helped us simplify the models and better understand which parameters should be refined to further improve model performance [36]. We trained the learner with ground PM_2.5_ measurements as the dependent variable. Independent variables included Aqua and Terra AOD, the day of the year, meteorological fields (precipitation, surface albedo, latent heat flux, surface evaporation, planetary boundary layer height, relative humidity, specific humidity, surface pressure, surface skin temperature, surface incident shortwave flux, surface velocity scale, air temperature, eastward wind component, northward wind component) and land use parameters (population density, land cover and elevation). Then we used trained models and predictor variables to predict daily PM_2.5_ in each 5 km × 5 km grid cell. We developed two separate random forest models for reference semester and pandemic semester. Random forest models are a combination of tree predictors, and each tree is constructed using the best split for each node among a subset of predictors randomly chosen at that node [37,38]. Both models had the same predictor variables while differing in their variable important rankings. By comparing the results with different settings, we set m_try_ and n_tree_ as 7 and 500, respectively, to achieve the best prediction accuracy. Highly correlated variables and predictors with low importance rankings were eliminated from the model. The final PM_2.5_ prediction model is expressed as:

$$\text{PM}2.5_{\text{st}}={\text{f (Aqua and Terra AOD}}_{\text{st}},\text{surface albedost},\text{latent heat fluxst},{\text{surface evaporation}}_{\text{st}},\text{planetary}\;{\text{boundary layer height}}_{\text{st}},{\text{surface incident shortwave flux}}_{\text{st}},{\text{surface velocity scale}}_{\text{st}},\text{eastward wind}\;{\text{component}}_{\text{st}},{\text{northward wind component}}_{\text{st}},{\text{surface pressure}}_{\text{st}},{\text{air temperature}}_{\text{st}},{\text{skin temperature}}_{\text{st}},\;{\text{precipitation}}_{\text{st}},{\text{relative humidity}}_{\text{st}},{\text{specific humidity}}_{\text{st}},\text{land covers},{\text{population density}}_{s},{\text{elevation}}_{s}).$$

where s represents the location of a grid cell and t represents the day of an observation. Variables with low importance values were discarded from the models following the variable selection strategy [37].

To assess model prediction performance, we applied 10-fold cross-validation techniques. The reference year model and pandemic year model were validated separately. Each model training dataset was randomly split into 10 groups with 10% of the total data in each group. During each round of cross-validation, we used nine groups to fit the random forest models and used the remaining one group as testing samples. The validation process was repeated 10 times until every group was tested. We calculated various statistical indicators such as the coefficient of determination (R^2^), mean absolute percentage error (MAPE) and root mean square error (RMSE) between cross-validated predictions and observations. A comparison was conducted between the CV and model fitting statistics to test for potential model overfitting. All statistical analyses were performed using cross_var_score, DecisionTreeRegressor and RandomForestRegressor libraries in Python software, version 3.7.6.

## 3. Results

### 3.1. Descriptive Statistics

The reference semester model dataset had 181 sample days with 61 days, 59 days and 61 days in subperiods 1, 2 and 3, respectively. The pandemic semester dataset had 182 sample days with 61 days, 60 days and 61 days in every subperiod. As shown in Table S2, the mean PM_2.5_ concentrations for the reference semester were 41.30 μg/m^3^ and 36.52 μg/m^3^ for the pandemic semester.

During the reference year, the mean PM_2.5_ concentration in subperiod 2 (45.54 μg/m^3^) was noticeably higher than subperiod 1 (42.15 μg/m^3^) and 3 (36.22 μg/m^3^). The PM_2.5_ concentrations increased during subperiod 2 probably because of the Chinese New Year migration and celebration activities such as firecracker burning [39]. In the pandemic semester model dataset, the mean PM_2.5_ concentration during subperiod 2 (36.88 μg/m^3^) was comparable to the other two periods (36.52 and 36.88 μg/m^3^).

### 3.2. Model Performance and Variable Importance

**Figure 3.**
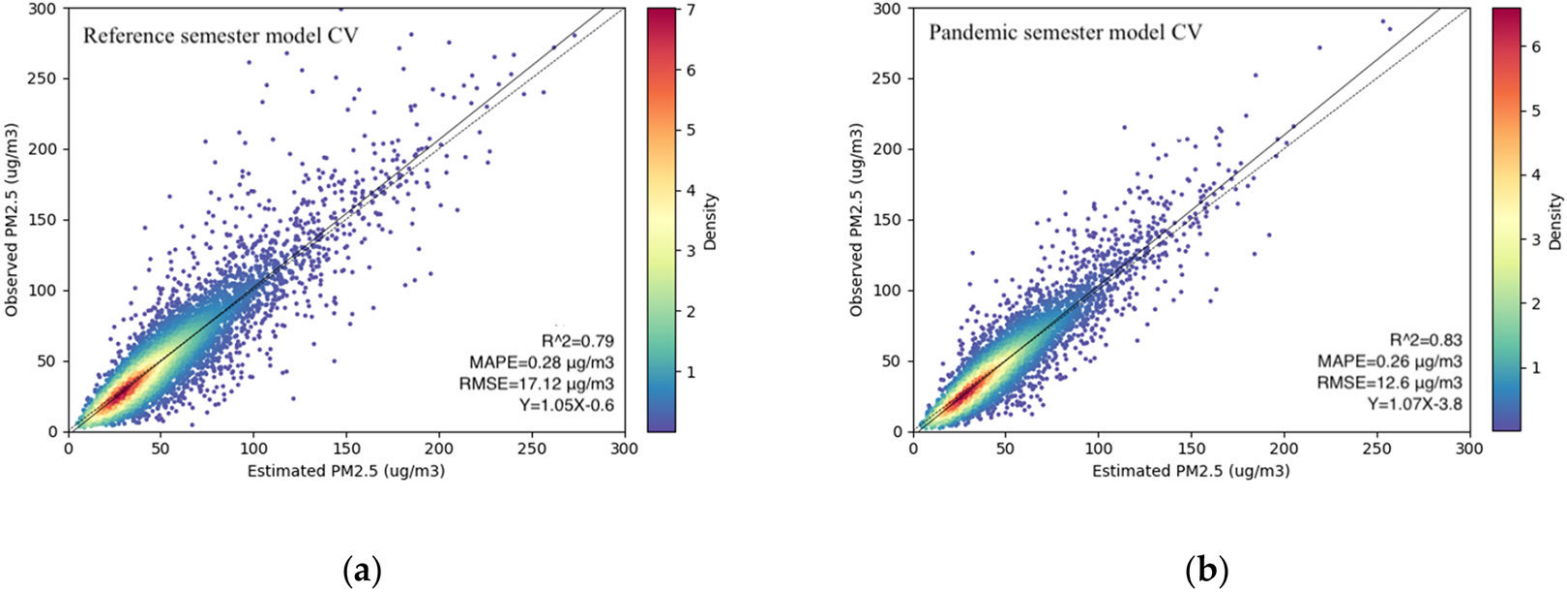
(**a**) Density plot of ground and monitored PM_2.5_ measurements in μg/m^3^ based on the 10-fold cross-validation of the reference semester model; (**b**) density plot of ground and monitored PM_2.5_ measurements in μg/m^3^ based on the 10-fold cross-validation of the pandemic semester model.

The 10-fold cross-validation results for the reference semester model and pandemic semester model are presented in Figure 3. For the reference semester model, the cross-validated (CV) R^2^ between fitted and observed PM_2.5_ concentrations was 0.79. The MAPE and RMSE were 0.28 μg/m^3^ and 17.55 μg/m^3^, respectively. For the pandemic semester model, the CV R^2^ increased to 0.83. The MAPE and RMSE decreased to 0.26 μg/m^3^ and 13.48 μg/m^3^, respectively, demonstrating a good agreement between CV predictions and ground observations. Figure 3 also shows that both models underestimated PM_2.5_ concentrations at high concentration levels. The random forest algorithm presented the relative importance of predictor variable in the two prediction models by calculating %IncMSE. %IncMSE is the increase in mean square error of predictions (estimated with out-of-bag-CV) as a result of variable j being permuted (values randomly shuffled). A higher %IncMSE indicates greater importance of a variable in the prediction. For the reference semester model, the AOD parameter ranked highest in terms of importance. Meteorological parameters such as surface incident shortwave flux, planetary boundary layer height and latent heat flux, as well as the elevation, also ranked high. For the pandemic semester model, the Aqua and Terra AOD and meteorological variables still ranked highest but land use parameters (population density, elevation and land cover) ranked low in terms of importance.

### 3.3. PM_2.5_ Predictions

**Figure 4.**
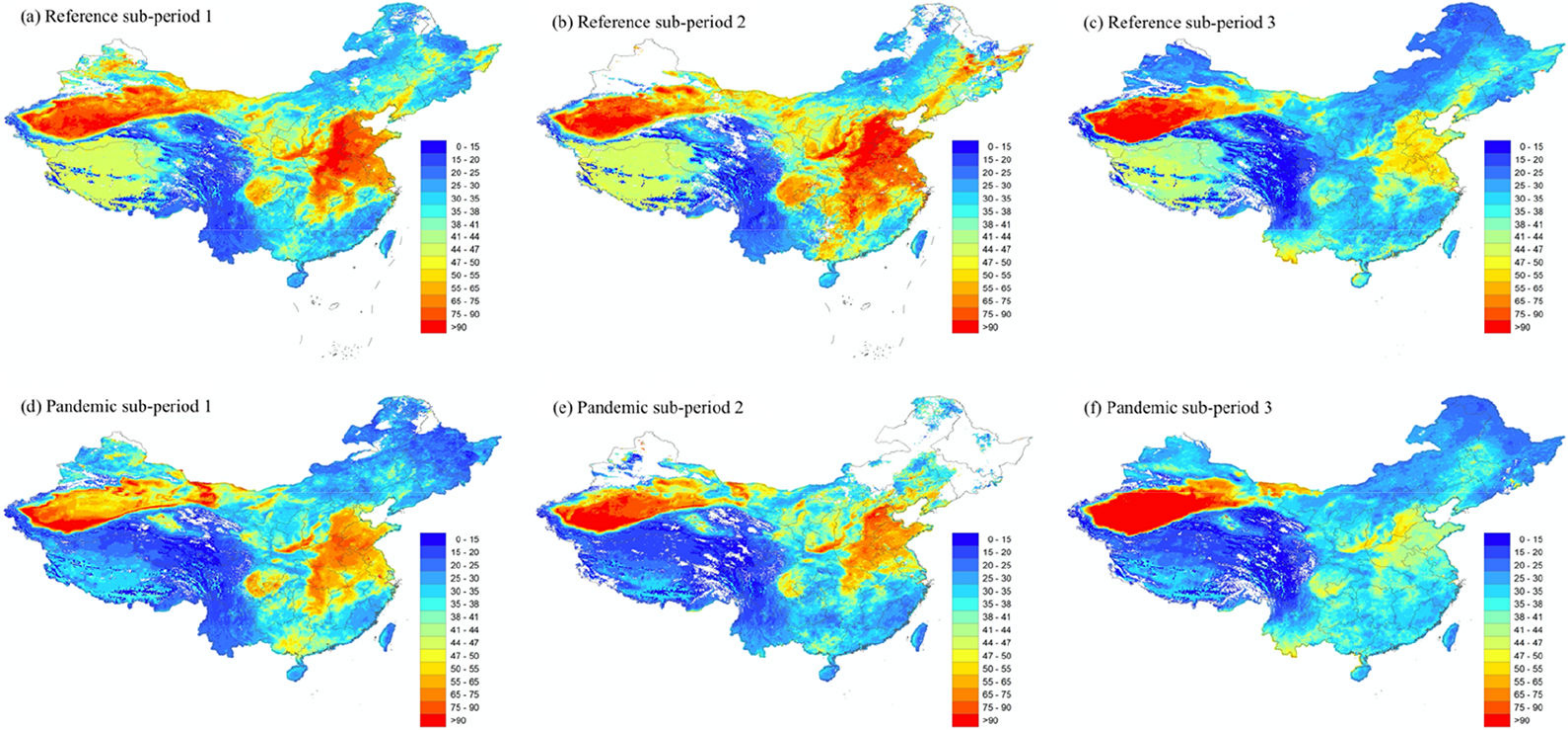
(**a**) Average PM_2.5_ distribution in the reference sub-period 1; (**b**) Average PM_2.5_ distribution in the reference subperiod 2; (**c**) Average PM_2.5_ distribution in the reference sub-period 3; (**d**) Average PM_2.5_ distribution in the pandemic sub-period 1; (**e**) Average PM_2.5_ distribution in the pandemic sub-period 2; (**f**) Average PM_2.5_ distribution in the pandemic sub-period 3. ArcGIS was used (version 10.7.1).

The spatial distribution of mean PM_2.5_ predictions during subperiods 1, 2 and 3 by the reference semester model and pandemic semester model is presented in Figure 4. The reference semester model had a spatial coverage of 95% for subperiod 1, 87% for subperiod 2 and 96% for subperiod 3. The mean PM_2.5_ concentrations in every subperiod (1, 2 and 3) were 41.25 μg/m^3^, 45.54 μg/m^3^ and 36.22 μg/m^3^, respectively. For the reference semester model, maps showed similar spatial patterns of PM_2.5_ concentrations in subperiods 1 and 2. The mean PM_2.5_ distribution maps during these two periods (Figure 4A,B) show regions with elevated PM_2.5_ levels in the North China Plain, including Beijing, Tianjin, Hebei province and Henan province, as well as the NYRD region. The NYRD region had the highest PM_2.5_ concentrations during subperiods 1 and 2; 68.72 μg/m^3^ and 74.35 μg/m^3^, respectively, compared to other regions (Table 1). The rapid urbanization, high population density and local economic growth were main driving forces of high PM_2.5_ concentrations in East China [40,41]. There were also some hotspots in the Sichuan Basin, especially in two megacities: Chengdu and Chongqing [42]. The Sichuan Basin is completely encircled by high mountains and plateaus. It is also characterized by persistently high relative humidity as well as low wind speeds [43,44]. The discharge of anthropogenic pollutants in combination with the special topography and meteorological conditions limits the diffusion of pollutants in this region [42,45,46]. In addition, high levels of PM_2.5_ pollution were found in the northwestern region, especially in the southern Xinjiang Autonomous Region where the Taklamakan Desert covers 60% of this region. The mean PM_2.5_ concentrations during subperiods 1 and 2 in the northwestern region were 57.45 μg/m^3^ and 60.66 μg/m^3^, respectively. The accumulation of dust particles in the winter contributed to the high level of PM_2.5_ pollution in this region [35,47]. During subperiod 3 (Figure 4C), PM_2.5_ concentrations stayed high in the northwestern region (56.29 μg/m^3^) but substantially decreased in the NYRD region (44.58 μg/m^3^).

For the pandemic semester model, the spatial coverage for mean PM_2.5_ concentrations during subperiods 1, 2 and 3 were 96%, 79% and 96%, respectively. The mean PM_2.5_ distribution map for subperiod 1 (Figure 4D) indicates high PM_2.5_ concentrations in the North China Plain, Yangtze River Delta, Sichuan Basin and northwestern region. During subperiod 2 and 3 (Figure 4E,F), there were fewer hotspots in eastern China and Sichuan Basin while PM_2.5_ concentrations significantly increased in the northwestern region. Then, mean PM_2.5_ concentrations in the northwestern region in each subperiod (1, 2 and 3) were 46.74 μg/m^3^, 54.18 μg/m^3^ and 68.67 μg/m^3^, respectively.

## 4. Discussion

Our machine learning method had strong potential to estimate PM_2.5_ concentrations and presented spatial and temporal variability during the COVID-19 outbreak. Our model demonstrated high prediction accuracy on a national scale and yielded a similar CV R^2^ to previous studies conducted in China [31,46]. Additionally, our study domain is geographically broad, which allowed us to explore spatial variations across China. Many studies examining changes in PM_2.5_ pollution during the pandemic relied solely on ground measurements, which failed to provide comprehensive spatial coverage, especially in suburban and rural regions. As a result, previous studies could only focus on certain cities or one city-cluster region [8,23].

Our reference semester model showed high PM_2.5_ concentrations in the North China Plain, northern Yangtze River Delta, Sichuan Basin and Xinjiang Autonomous Region. Overall, the levels of PM_2.5_ pollution were higher in the northern regions than in the southern regions. Our predictions showed similar spatial distributions and variations compared with other studies in these regions [27,46,48-51]. The intensive human activities (i.e., industrial activities, fossil fuel combustion and agricultural waste burning) and unfavorable meteorological conditions (low boundary layer height and weak wind) led to high PM_2.5_ concentrations in the North China Plain [41,46,51]. The main reasons for the serious PM_2.5_ pollution in the Yangtze River Delta were high population density and rapid urbanization [40]. The Sichuan Basin had high PM_2.5_ pollution due to its unique topography. Persistent temperature inversion and stagnant air circulation always occurs in this region [51]. Additionally, the dust storms in the desert region led to serious PM_2.5_ pollution in Xinjiang Autonomous Region [47]. Low PM_2.5_ pollution occurred in the northeastern region characterized by its dense vegetation cover. The southern region generally had low PM_2.5_ concentrations because it benefits from its favorable meteorological conditions (i.e., high precipitation and southerly flow) for atmospheric dispersion [52].

**Figure 5.**
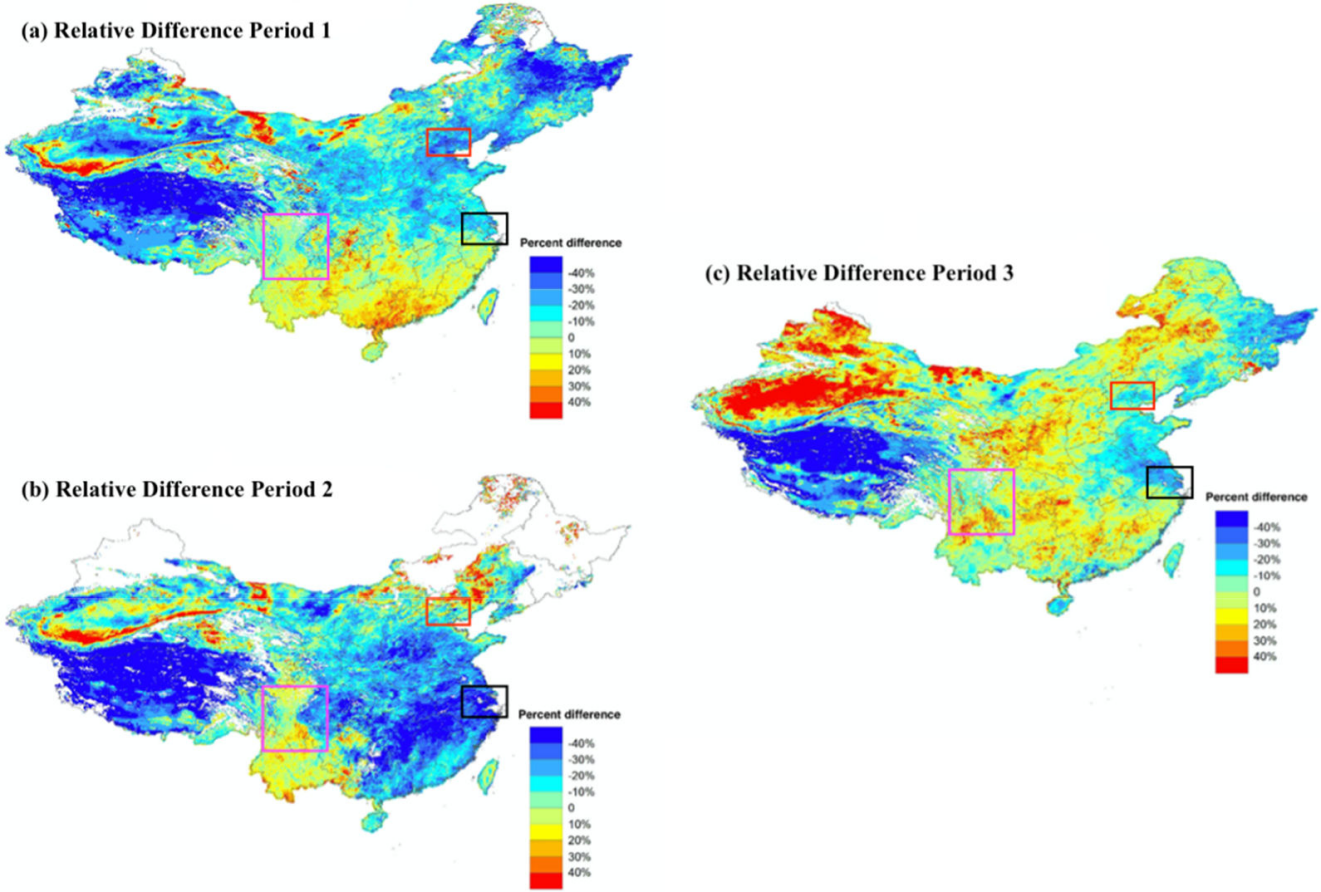
(**a**) Estimated PM_2.5_ change rates between the reference semester model and pandemic semester model predictions in sub-period 1; (**b**) Estimated PM_2.5_ change rates between the reference semester model and pandemic semester model predictions in sub-period 2; (**c**) Estimated PM_2.5_ change rates between the reference semester model and pandemic semester model predictions in sub-period 3. Beijing–Tianjin–Hebei Region, Yangtze River Delta and Sichuan Basin are marked in red, black and purple, respectively.

Our model predictions allowed us to explore the impact of COVID-19 on PM_2.5_ levels during the pandemic semester. PM_2.5_ levels were lowered by 4.8 μg/m^3^ during the pandemic semester as compared to the reference semester. We also calculated the relative difference between the reference semester model and semester year model predictions. Compared with the reference semester, PM_2.5_ levels in pandemic subperiods 1 and 2 decreased by 13% and 18% but increased slightly by 0.48% in subperiod 3 (Figure 5). During the pandemic subperiod 1, COVID-19 transmissibility had not been confirmed and no control measure had been implemented. PM_2.5_ concentrations decreased most in Northeast, Northwest and Qinghai–Tibet regions by 18%, 17% and 15%, respectively (Table 2). The decrease of PM_2.5_ levels in these regions of low population density was likely due to favorable meteorological conditions. China meteorological administration observed a significant increase in precipitation in Tibet and denser vegetation cover in the Northeast region (http://www.cma.gov.cn, accessed on 20 March 2021). The increased green space was able to regulate microclimatic conditions and reduce pollutants through filtration [53-55]. During subperiod 2, a significantly greater reduction in PM_2.5_ levels (18%, *p* < 0.05) was observed due to the COVID-19 outbreak, when lockdown and stringent traffic restrictions were implemented by the governments. PM_2.5_ levels in the Southeast region decreased most by 31%, followed by NYRD (29%) and PRD (24%). Yangtze River Delta and Pearl River Delta were major economic city-clusters in China. As they entered Level I public health response period (24 January–25 February), cities reduced the number of people and vehicles in public places and closed all industrial enterprises, construction sites and recreational operations [22]. Other studies focused on these two regions showed similar results. Li et al. (2020) found concentrations of PM_2.5_ decreased by 31.8% during the Level I period in the NYRD region compared with 2019 [22]. He et al. (2020) confirmed a reduction in the AQI around 5–10 points, converted to a reduction in PM_2.5_ around 1.2–2.4 μg/m^3^, in Southern China during the lockdown period relative to the previous year [9]. During this period, hotspots of PM_2.5_ were observed in Beijing–Tianjin–Hebei Region. The increase of PM_2.5_ levels in this region was contrary to the overall decreasing trend in the North region by 12%. Other studies that conducted atmospheric and transport model simulations in Beijing–Tianjin–Hebei Region showed similar PM_2.5_ concentration patterns during this period. Le et al. (2020) observed severe haze events in Beijing during the outbreak period and increased mean surface PM_2.5_ by 55.1% compared to the same period of 2015 to 2019 [56]. Unfavorable meteorological conditions such as low wind speed and high relative humidity in BTH (Beijing-Tianjin-Hebei) Region might explain increased PM_2.5_ levels [56-58]. During pandemic subperiod 3, cities with low risk of COVID-19 infection started to reopen and most activities entered into operation. Compared with the pandemic year period 2, PM_2.5_ levels statistically increased in the Southeast, Northwest, PRD, NYRD and North regions. PM_2.5_ concentrations increased in the Northwest region due to frequent dust storms occurring in spring in the desert, semidesert and grassland areas [59]. The temporal variability in other regions could be explained by increased human activities and industrial emissions in Yangtze River Delta, Pearl River Delta and North China Plain after the reopen. During this period, citizens were allowed to travel locally with health code and protection measures; commercial and industrial enterprises were allowed to resume work.

We were able to compare changes of PM_2.5_ levels in different land cover types. As we observed an overall decline of PM_2.5_ concentrations, urban areas had a larger reduction than in rural areas during the COVID-19 outbreak (Table 2). Several reasons could explain this disparity. First, the mass human migration during the Spring Festival travel led to the change of population distribution patterns in China. There was a significant reduction in population density in urban areas during the holiday period [60]. Combining with the COVID-19 control measures, the greater reduction in human activities in urban areas contributed to greater decrease in PM_2.5_ levels. Secondly, the proportion of bulk coal heating users increased in the rural areas due to the return of migrant workers and the lack of central heating. The increasing emissions may have mitigated reductions resulting from COVID-19 control measures. Moreover, life-essential industrial facilities such as power plants are located in rural areas and stayed in operation during the COVID-19 outbreak, while other industrial facilities and entertainment operations were closed in urban areas.

One limitation of this study is the incomplete spatial coverage due to cloud and snow cover, especially in northeastern China, which may introduce region-specific sampling biases when estimating mean PM_2.5_ levels in each period. We will address this issue in the future with a gap-filling method. Overall, we have a high spatial coverage and these missing values will not significantly alter our results. Another limitation of this study is the uneven distribution of ground monitoring measurements across the study domain. There are fewer monitoring locations in the Northwest and Qinghai–Tibet compared to other regions. Although our models reached high prediction accuracy, the lack of ground measurements for model training possibly influences the model performance in these two regions. We will address this issue in future research by fitting separate models in every cluster.

## 5. Conclusions

We developed a machine learning method with satellite-derived data as major predictor variables to provide spatiotemporally resolved daily PM_2.5_ estimates (reference semester model: CV R^2^ = 0.79, and pandemic semester model: CV R^2^ = 0.83). Our results show that the PM_2.5_ levels were lowered by 4.8 μg/m^3^ during the pandemic semester compared to the reference semester. COVID-19 control measures implemented during subperiod 2 caused significant reduction in PM_2.5_ levels by 18%. The Southeast region decreased most by 31% and the urban areas decreased more than rural areas. Though PM_2.5_ concentrations dropped significantly during the COVID-19 lockdown, the national average was still three times higher than safety levels suggested by the World Health Organization (10 μg/m^3^ for the annual mean). Our paper is useful for future research to understand the full implications of this unprecedented event and is informative with regards to more stringent air pollution regulations. Our PM_2.5_ predictions can be used to calculate the decreased disease burden resulting from PM_2.5_ pollution during the COVID-19 pandemic.

## Supplementary Material

Li supplement

## Figures and Tables

**Table 1.** Summary statistics of PM_2.5_ predictions by cluster during modeling periods (μg/m^3^).

	Period 1	Period 2	Period 3
**Reference semester**			
North	47.10	52.41	33.69
Northwest	57.45	60.66	56.29
Northeast	33.65	37.67	28.67
Qinghai–Tibet	33.55	33.97	30.25
NYRD	68.72	74.35	44.58
Southeast	35.23	45.25	31.84
PRD	36.55	46.46	33.39
**Pandemic semester**			
North	40.46	44.53	35.20
Northwest	46.72	54.18	68.67
Northeast	26.73	37.77	27.92
Qinghai–Tibet	27.39	24.76	26.08
NYRD	57.52	51.80	39.48
Southeast	35.45	29.46	32.38
PRD	41.20	32.84	34.51

**Table 2.** Estimated PM_2.5_ change rates by region and by land type during modeling periods (%).

Region	Period 1	Period 2	Period 3
North	−12.77	−12.68	5.45
Northwest	−16.91	−9.03	20.25
Northeast	−18.42	7.03	−0.16
Qinghai–Tibet	−15.24	−21.5	−10.08
NYRD	−14.56	−29.39	−9.48
Southeast	2.63	−31.05	3.14
PRD	13.92	−23.8	5.35
Urban	−13.49	−25.12	−7.33
Rural	−9.78	−19.68	1.22

## Data Availability

Not applicable.

## References

[1] Zhu N; Zhang D; Wang W; Li X; Yang B; Song J; Zhao X; Huang B; Shi W; Lu R A novel coronavirus from patients with pneumonia in China, 2019. N. Engl. J. Med 2020, 382, 727–733. 31978945 10.1056/NEJMoa2001017 PMC7092803

[2] Yuen K-S; Ye Z-W; Fung S-Y; Chan C-P; Jin D-Y SARS-CoV-2 and COVID-19: The most important research questions. Cell Biosci. 2020, 10, 1–5. 32190290 10.1186/s13578-020-00404-4 PMC7074995

[3] Zhou P; Yang X-L; Wang X-G; Hu B; Zhang L; Zhang W; Si H-R; Zhu Y; Li B; Huang C-L A pneumonia outbreak associated with a new coronavirus of probable bat origin. Nature 2020, 579, 270–273. 32015507 10.1038/s41586-020-2012-7 PMC7095418

[4] Shereen MA; Khan S; Kazmi A; Bashir N; Siddique R COVID-19 infection: Origin, transmission, and characteristics of human coronaviruses. J. Adv. Res 2020, 24, 91–98. 32257431 10.1016/j.jare.2020.03.005 PMC7113610

[5] Wu F; Zhao S; Yu B; Chen Y-M; Wang W; Song Z-G; Hu Y; Tao Z-W; Tian J-H; Pei Y-Y A new coronavirus associated with human respiratory disease in China. Nature 2020, 579, 265–269. 32015508 10.1038/s41586-020-2008-3 PMC7094943

[6] Leung K; Wu JT; Liu D; Leung GM First-wave COVID-19 transmissibility and severity in China outside Hubei after control measures, and second-wave scenario planning: A modelling impact assessment. Lancet 2020, 395, 1382–1393. 32277878 10.1016/S0140-6736(20)30746-7 PMC7195331

[7] Tian H; Liu Y; Li Y; Wu C-H; Chen B; Kraemer MU; Li B; Cai J; Xu B; Yang Q An investigation of transmission control measures during the first 50 days of the COVID-19 epidemic in China. Science 2020, 368, 638–642. 32234804 10.1126/science.abb6105 PMC7164389

[8] Lian X; Huang J; Huang R; Liu C; Wang L; Zhang T Impact of city lockdown on the air quality of COVID-19-hit of Wuhan city. Sci. Total Environ 2020, 742, 140556. 32634686 10.1016/j.scitotenv.2020.140556 PMC7326389

[9] He G; Pan Y; Tanaka T The short-term impacts of COVID-19 lockdown on urban air pollution in China. Nat. Sustain 2020, 3, 1005–1011.

[10] Di Q; Wang Y; Zanobetti A; Wang Y; Koutrakis P; Choirat C; Dominici F; Schwartz JD Air pollution and mortality in the Medicare population. N. Engl. J. Med 2017, 376, 2513–2522. 28657878 10.1056/NEJMoa1702747 PMC5766848

[11] Haikerwal A; Akram M; del Monaco A; Smith K; Sim MR; Meyer M; Tonkin AM; Abramson MJ; Dennekamp M Impact of fine particulate matter (PM2.5) exposure during wildfires on cardiovascular health outcomes. J. Am. Heart Assoc 2015, 4, e001653. 26178402 10.1161/JAHA.114.001653 PMC4608063

[12] Lee B-J; Kim B; Lee K Air pollution exposure and cardiovascular disease. Toxicol. Res 2014, 30, 71–75. 25071915 10.5487/TR.2014.30.2.071 PMC4112067

[13] Pope CA III; Burnett RT; Thurston GD; Thun MJ; Calle EE; Krewski D; Godleski JJ Cardiovascular mortality and long-term exposure to particulate air pollution: Epidemiological evidence of general pathophysiological pathways of disease. Circulation 2004, 109, 71–77. 14676145 10.1161/01.CIR.0000108927.80044.7F

[14] Dominici F; Peng RD; Bell ML; Pham L; McDermott A; Zeger SL; Samet JM Fine particulate air pollution and hospital admission for cardiovascular and respiratory diseases. JAMA 2006, 295, 1127–1134. 16522832 10.1001/jama.295.10.1127 PMC3543154

[15] Kloog I; Melly SJ; Ridgway WL; Coull BA; Schwartz J Using new satellite based exposure methods to study the association between pregnancy PM2.5 exposure, premature birth and birth weight in Massachusetts. Environ. Health 2012, 11, 1–8. 22709681 10.1186/1476-069X-11-40 PMC3464884

[16] Zhu X; Liu Y; Chen Y; Yao C; Che Z; Cao J Maternal exposure to fine particulate matter (PM2.5) and pregnancy outcomes: A meta-analysis. Environ. Sci. Pollut. Res 2015, 22, 3383–3396. 10.1007/s11356-014-3458-7 25163563

[17] Feng C; Li J; Sun W; Zhang Y; Wang Q Impact of ambient fine particulate matter (PM2.5) exposure on the risk of influenza-like-illness: A time-series analysis in Beijing, China. Environ. Health 2016, 15, 17. 26864833 10.1186/s12940-016-0115-2 PMC4750357

[18] Wu X; Nethery RC; Sabath BM; Braun D; Dominici F Exposure to air pollution and COVID-19 mortality in the United States. MedRxiv 2020, 6, eabd4049. 10.1126/sciadv.abd4049 PMC7673673 33148655

[19] Liang F; Xiao Q; Huang K; Yang X; Liu F; Li J; Lu X; Liu Y; Gu D The 17-y spatiotemporal trend of PM2.5and its mortality burden in China. Proc. Natl. Acad. Sci. USA 2020, 117, 25601. 32958653 10.1073/pnas.1919641117 PMC7568266

[20] Berman JD; Ebisu K Changes in US air pollution during the COVID-19 pandemic. Sci. Total Environ 2020, 739, 139864. 32512381 10.1016/j.scitotenv.2020.139864 PMC7442629

[21] Zangari S; Hill DT; Charette AT; Mirowsky JE Air quality changes in New York City during the COVID-19 pandemic. Sci. Total Environ 2020, 742, 140496. 32640401 10.1016/j.scitotenv.2020.140496 PMC7314691

[22] Li L; Li Q; Huang L; Wang Q; Zhu A; Xu J; Liu Z; Li H; Shi L; Li R Air quality changes during the COVID-19 lockdown over the Yangtze River Delta Region: An insight into the impact of human activity pattern changes on air pollution variation. Sci. Total Environ 2020, 732, 139282. 32413621 10.1016/j.scitotenv.2020.139282 PMC7211667

[23] He G; Pan Y; Tanaka T COVID-19, City Lockdowns, and Air Pollution: Evidence from China. medRxiv 2020.

[24] Donkelaar AV; Martin RV; Brauer M; Kahn R; Levy R; Verduzco C; Villeneuve PJ Global Estimates of Ambient Fine Particulate Matter Concentrations from Satellite-Based Aerosol Optical Depth: Development and Application. Environ. Health Perspect 2010, 118, 847–855. 20519161 10.1289/ehp.0901623 PMC2898863

[25] Chudnovsky AA; Koutrakis P; Kloog I; Melly S; Nordio F; Lyapustin A; Wang Y; Schwartz J Fine particulate matter predictions using high resolution Aerosol Optical Depth (AOD) retrievals. Atmos. Environ 2014, 89, 189–198. 10.1016/j.atmosenv.2014.07.014 PMC5621749 28966552

[26] Hu X; Waller LA; Lyapustin A; Wang Y; Al-Hamdan MZ; Crosson WL; Estes MG; Estes SM; Quattrochi DA; Puttaswamy SJ Estimating ground-level PM2.5 concentrations in the Southeastern United States using MAIAC AOD retrievals and a two-stage model. Remote Sens. Environ 2014, 140, 220.

[27] Ma Z; Hu X; Sayer AM; Levy R; Zhang Q; Xue Y; Tong S; Bi J; Huang L; Liu Y Satellite-Based Spatiotemporal Trends in PM2.5 Concentrations: China, 2004–2013. Environ. Health Perspect 2016, 124, 184–192. 26220256 10.1289/ehp.1409481 PMC4749081

[28] Lyapustin A; Wang Y; Korkin S; Huang D MODIS Collection 6 MAIAC algorithm. Atmos. Meas. Tech 2018, 11, 5741–5765.

[29] Green M; Kondragunta S; Ciren P; Xu C Comparison of GOES and MODIS aerosol optical depth (AOD) to aerosol robotic network (AERONET) AOD and IMPROVE PM2.5 mass at Bondville, Illinois. J. Air Waste Manag. Assoc 2009, 59, 1082–1091. 19785275 10.3155/1047-3289.59.9.1082

[30] Puttaswamy SJ; Nguyen HM; Braverman A; Hu X; Liu Y Statistical data fusion of multi-sensor AOD over the Continental United States. Geocarto Int. 2013, 29, 48–64.

[31] Xiao Q; Chang HH; Geng G; Liu Y An ensemble machine-learning model to predict historical PM2.5 concentrations in China from satellite data. Environ. Sci. Technol 2018, 52, 13260–13269. 30354085 10.1021/acs.est.8b02917

[32] Song N; Ma J; Yu Y; Yang Z; Li Y New observations on PAH pollution in old heavy industry cities in northeastern China. Environ. Pollut 2015, 205, 415–423. 26189045 10.1016/j.envpol.2015.07.005

[33] Kong S; Shi J; Lu B; Qiu W; Zhang B; Peng Y; Zhang B; Bai Z Characterization of PAHs within PM10 fraction for ashes from coke production, iron smelt, heating station and power plant stacks in Liaoning Province, China. Atmos. Environ 2011, 45, 3777–3785.

[34] Sun L; Zang SY; Sun HJ Sources and history of PAHs in lake sediments from oil-producing and industrial areas, northeast China. Int. J. Environ. Sci. Technol 2014, 11, 2051–2060.

[35] Chen Z; Cheng S; Li J; Guo X; Wang W; Chen D Relationship between atmospheric pollution processes and synoptic pressure patterns in northern China. Atmos. Environ 2008, 42, 6078–6087.

[36] Huang K; Bi J; Meng X; Geng G; Lyapustin A; Lane KJ; Gu D; Kinney PL; Liu Y Estimating daily PM2.5 concentrations in New York City at the neighborhood-scale: Implications for integrating non-regulatory measurements. Sci. Total Environ 2019, 697, 134094. 32380602 10.1016/j.scitotenv.2019.134094

[37] Hu X; Belle JH; Meng X; Wildani A; Waller LA; Strickland MJ; Liu Y Estimating PM2.5 concentrations in the conterminous United States using the random forest approach. Environ. Sci. Technol 2017, 51, 6936–6944. 28534414 10.1021/acs.est.7b01210

[38] Breiman L Random Forests. Mach. Learn 2001, 45, 5–32.

[39] Ye C; Chen R; Chen M The impacts of Chinese Nian culture on air pollution. J. Clean. Prod 2016, 112, 1740–1745.

[40] Lin G; Fu J; Jiang D; Hu W; Dong D; Huang Y; Zhao M Spatio-temporal variation of PM2.5 concentrations and their relationship with geographic and socioeconomic factors in China. Int. J. Environ. Res. Public Health 2014, 11, 173–186. 10.3390/ijerph110100173 PMC3924439 24362546

[41] Li T; Shen H; Zeng C; Yuan Q; Zhang L Point-surface fusion of station measurements and satellite observations for mapping PM2.5 distribution in China: Methods and assessment. Atmos. Environ 2017, 152, 477–489.

[42] Wang H; Tian M; Chen Y; Shi G; Liu Y; Yang F; Zhang L; Deng L; Yu J; Chao P Seasonal characteristics, formation mechanisms and source origins of PM2.5 in two megacities in Sichuan Basin, China. Atmos. Chem. Phys. 2018, 18, 865.

[43] Guo M; Cai X; Song Y Characteristics of low wind-speed meteorology in China. Acta Sci. Nat. Univ. Pekin. 2016, 52, 219–226.

[44] Chen Y; Xie S-D Long-term trends and characteristics of visibility in two megacities in southwest China: Chengdu and Chongqing. J. Air Waste Manag. Assoc 2013, 63, 1058–1069. 24151681 10.1080/10962247.2013.791348

[45] Wang X; Dickinson RE; Su L; Zhou C; Wang K PM2.5 pollution in China and how it has been exacerbated by terrain and meteorological conditions. Bull. Am. Meteorol. Soc 2018, 99, 105–119.

[46] Wei J; Huang W; Li Z; Xue W; Peng Y; Sun L; Cribb M Estimating 1-km-resolution PM2.5 concentrations across China using the space-time random forest approach. Remote Sens. Environ 2019, 231, 111221.

[47] Fang X; Zou B; Liu X; Sternberg T; Zhai L Satellite-based ground PM2.5 estimation using timely structure adaptive modeling. Remote Sens. Environ 2016, 186, 152–163.

[48] Ma Z; Hu X; Huang L; Bi J; Liu Y Estimating Ground-Level PM2.5 in China Using Satellite Remote Sensing. Environ. Sci. Technol 2014, 48, 7436–7444. 24901806 10.1021/es5009399

[49] He Q; Huang B Satellite-based mapping of daily high-resolution ground PM2.5 in China via space-time regression modeling. Remote Sens. Environ 2018, 206, 72–83.

[50] Zhang M; Wang X; Chen J; Cheng T; Wang T; Yang X; Gong Y; Geng F; Chen C Physical characterization of aerosol particles during the Chinese New Year’s firework events. Atmos. Environ 2010, 44, 5191–5198.

[51] You W; Zang Z; Zhang L; Li Y; Pan X; Wang W National-Scale Estimates of Ground-Level PM2.5 Concentration in China Using Geographically Weighted Regression Based on 3 km Resolution MODIS AOD. Remote Sens. 2016, 8, 184. 10.1007/s11356-015-6027-9 26780051

[52] Zhai S; Jacob DJ; Wang X; Shen L; Li K; Zhang Y; Gui K; Zhao T; Liao H Fine particulate matter (PM2.5) trends in China, 2013–2018: Separating contributions from anthropogenic emissions and meteorology. Atmos. Chem. Phys 2019, 19, 11031–11041.

[53] Hwang H-J; Yook S-J; Ahn K-H Experimental investigation of submicron and ultrafine soot particle removal by tree leaves. Atmos. Environ 2011, 45, 6987–6994.

[54] Lafortezza R; Carrus G; Sanesi G; Davies C Benefits and well-being perceived by people visiting green spaces in periods of heat stress. Urban For. Urban Green 2009, 8, 97–108.

[55] Chen J; Zhu L; Fan P; Tian L; Lafortezza R Do green spaces affect the spatiotemporal changes of PM2.5 in Nanjing? Ecol. Process 2016, 5, 7. 27570725 10.1186/s13717-016-0052-6 PMC4986350

[56] Le T; Wang Y; Liu L; Yang J; Yung YL; Li G; Seinfeld JH Unexpected air pollution with marked emission reductions during the COVID-19 outbreak in China. Science 2020, 369, 702–706. 32554754 10.1126/science.abb7431 PMC7402623

[57] Zhao N; Wang G; Li G; Lang J; Zhang H Air pollution episodes during the COVID-19 outbreak in the Beijing—Tianjin—Hebei region of China: An insight into the transport pathways and source distribution. Environ. Pollut 2020, 267, 115617. 33254609 10.1016/j.envpol.2020.115617 PMC7477629

[58] Wang P; Chen K; Zhu S; Wang P; Zhang H Severe air pollution events not avoided by reduced anthropogenic activities during COVID-19 outbreak. Resour. Conserv. Recycl 2020, 158, 104814. 32300261 10.1016/j.resconrec.2020.104814 PMC7151380

[59] Zou XK; Zhai PM Relationship between vegetation coverage and spring dust storms over northern China. J. Geophys. Res. Atmos 2004, 109, D3.

[60] Hu M Visualizing the largest annual human migration during the Spring Festival travel season in China. Environ. Plan. A Econ. Space 2019, 51, 1618–1621.

