# Supplementary material for: Estimating the Impact of COVID-19 on the PM2.5 Levels in China with a Satellite-Driven Machine Learning Model
Source: Remote Sens (Basel). Author manuscript; Available in PMC 2021 Sep 20. (PMC8452231; doi:10.3390/rs13071351)
Supplement: Li supplement [file NIHMS1702349-supplement-Li_supplement.pdf]

Estimating the Impact of COVID-19 on the $PM_{2.5}$ Levels in China with A Satellite-Driven Machine Learning Model

Qiulun Li [1], Qingyang Zhu [1], Muwu Xu [1], Yu Zhao [2], KM Venkat Narayan [3] and Yang Liu [1,*]

[1] Gangarosa Department of Environmental Health, Rollins School of Public Health, Emory University, GA 30322, USA; qiulun.li@emory.edu (Q.L.); qingyang.zhu@emory.edu (Q.Z.); muwu.xu@emory.edu (M.X.)
[2] School of The Environment, Nanjing University, Nanjing 210023, China; yuzhao@nju.edu.cn (Y.Z.)
[3] Hubert Department of Global Health, Rollins School of Public Health, Emory University, GA 30322, USA; knaraya@emory.edu (KMV.N.)
* Correspondence: yang.liu@emory.edu

Table S1. Summary statistics of $PM_{2.5}$ predictions by cluster during modeling periods (μg/m[3]).

Source category	Data source	Variables	Resolution	
			Spatial	Temporal
$PM_{2.5}$	CNEMC	$PM_{2.5}$	Monitoring stations	hourly
	Hongkong EPD	$PM_{2.5}$	Monitoring stations	hourly
	Taiwan EPA	$PM_{2.5}$	Monitoring stations	hourly
AOD	MAIAC	Aqua and Terra AOD	1 km	daily
Meteorology	GEOS 5-FP	precipitation, surface albedo, latent heat flux, surface evaporation, planetary boundary layer height, relative humidity, specific humidity, surface pressure, surface skin temperature, surface incident shortwave flux, surface velocity scale, air temperature, eastward wind component, northward wind component	0.25° latitude × 0.3125° longitude	Hourly and 3-hourly
Land cover	ESA CCI	types of land cover	300 m	none
Population	LandScan	population density	1 km	none
Elevation	ASTER	Global Digital Elevation Model (GDEM) version 3	30 m	none

Table S2. summary statistics of $PM_{2.5}$ predictions, satellite AOD and major meteorological observations during modeling periods.

	Overall Mean	Period 1 (11/01 - 12/31) Mean	SD	Max	Min	Period 2 (1/01 - 2-28) Mean	SD	Max	Min	Period 3 (3/01 - 4/30) Mean	SD	Max	Min
Reference year		N = 61				N = 59				N = 61			
PM2.5 (μg/m^3)	41.30	42.15	19.26	199	9.22	45.54	21.61	261	6.61	36.22	20.44	293	6.68
Aqua and Terra AOD	0.2	0.16935	0.21	3.92	0.002	0.19	0.25	3.92	0.003	0.25	0.36	3.92	0.003
Albedo	0.25	0.25	0.12	0.83	0.064	0.28	0.14	0.85	0.063	0.23	0.12	0.84	0.057
EFLUX	68	54	56	1426	-26	50	50	1248	-7.21	101	86	910	-55
QV2M	3.281×10^{-3}	3.018×10^{-3}	2.89×10^{-3}	1.87×10^{-2}	9.89×10^{-5}	2.53×10^{-3}	2.36×10^{-3}	1.78×10^{-2}	9.04×10^{-5}	4.3×10^{-3}	3.75×10^{-3}	2.21×10^{-2}	2.73×10^{-4}
PBLH	1121	890	544	3690	55	933	637	3917	54	1540	711	4561	57
SWGDN	457	362	150	758	3.81	397	157	839	7.62	613	190	1041	26.09
RH	0.46	0.45	0.21	1	0.0098	0.52	0.21	1	0.15	0.41	0.22	1	0.031
Pandemic year		N = 61				N = 60				N = 61			
PM2.5 (μg/m^3)	36.52	35.81	17.62	317	4.42	36.88	20.10	342	4.57	36.88	28.54	352	4.20
Aqua and Terra AOD	0.23	0.18	0.21	3.92	0	0.21	0.27	3.92	0.005	0.3	0.42	3.92	0.004
Albedo	0.27	0.25	0.12	0.85	0.065	0.31	0.16	0.85	0.061	0.25	0.14	0.83	0.059
EFLUX	66	52	54	1402	-53	51	47	939	-31	95	79	1141	-16.09
QV2M	3.23×10^{-3}	2.9×10^{-3}	2.58×10^{-3}	1.9×10^{-2}	1.1×10^{-4}	2.79×10^{-3}	2.45×10^{-3}	1.74×10^{-2}	1.69×10^{-4}	3.99×10^{-3}	3.14×10^{-3}	1.98×10^{-2}	2.53×10^{-4}
PBLH	1070	946	578	3546	55	833	571	3521	55	1429	707	4736	61
SWGDN	465	373	150	772	10.32	411	169	868	9.49	610	213	1030	15.81
RH	0.49	0.47	0.21	1	0.019	0.56	0.23	1	0.03	0.43	0.24	1	0.011
